# LKB1 Dysregulation in Duchenne Muscular Dystrophy Models: Disease Specificity and Epigenetic Control by HDAC Inhibitors

**DOI:** 10.1111/nyas.70364

**Published:** 2026-08-20

**Authors:** Brigida Boccanegra, Lisamaura Tulimiero, Raffaella Quarta, Elena Conte, Simonetta Andrea Licandro, Alessandra Decio, Roberta Lenti, Alberto Ladisa, Giorgia Dinoi, Gaia Carbone, Letizia Claudione, Giulia Maria Camerino, Sabata Pierno, Paola Mantuano, Ornella Cappellari, Gianluca Fossati, Christian Steinkühler, Annamaria De Luca

**Affiliations:** ^1^ Department of Pharmacy – Drug Sciences University of Bari “Aldo Moro” Bari Italy; ^2^ Preclinical R&D Department Italfarmaco S.p.A. Milan Italy

**Keywords:** AMPK, animal models, Duchenne muscular dystrophy, epigenetic, histone deacetylases inhibitors, liver kinase B1, microRNAs

## Abstract

Efficient skeletal muscle contraction requires tight mechano‐metabolic coupling, a process regulated by AMP‐activated protein kinase (AMPK). Duchenne muscular dystrophy (DMD) is characterized by aberrant AMPK activation and disrupted metabolic signaling. This study investigates the expression and regulation of the LKB1–STRADα–MO25 heterotrimeric complex, the primary upstream activator of AMPK, in DMD models. We analyzed muscles from dystrophic mice (BL10 *mdx* and D2 *mdx*) and patient‐derived cells and found significant downregulation of the LKB1 complex across all disease stages in the DMD models, a defect not observed in an amyotrophic lateral sclerosis model. Treatment with the broad‐spectrum HDAC inhibitor vorinostat effectively restored LKB1 expression at both transcript and protein levels in D2 *mdx* mice. This restoration was mechanistically linked to downregulation of miR‐451, miR‐195, and miR‐17, which function as post‐transcriptional repressors of LKB1. Conversely, the selective HDAC1/2 inhibitor Rodin‐A increased *Lkb1* mRNA but failed to rescue protein levels or alter miRNA expression. Our data identify the axis LKB1–STRADα–MO25 as a critical regulatory node that is disrupted in DMD, but remains responsive to epigenetic modulation. These findings suggest that restoring LKB1 activity via HDAC inhibition or miRNA targeting may represent a therapeutic avenue to address dystrophic muscle dysfunction.

## Graphical abstract

Our study demonstrated that the LKB1–STRADα–MO25 complex was significantly downregulated in Duchenne muscular dystrophy (DMD) models, concurring with the disruption of mechano‐metabolic signaling. Treatment with the HDAC inhibitor, vorinostat, successfully restored LKB1 expression by suppressing specific repressor miRNAs. These findings identify the LKB1 axis as a critical, epigenetically modifiable node, suggesting that restoring its activity is a promising therapeutic strategy for DMD.

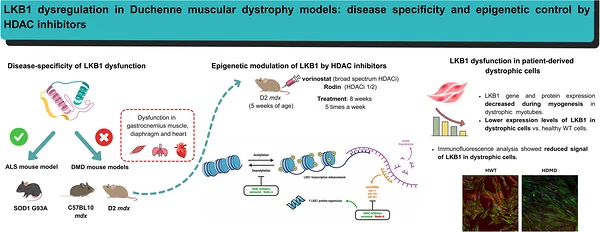

## 1 Introduction

The efficiency of skeletal muscle contraction relies on the precise coupling between mechanical input and metabolic processes to sustain force generation and energy homeostasis. This mechano‐metabolic coupling ensures that ATP production meets the high energy demands of muscle activity. A key player in this process is the AMP‐activated protein kinase (AMPK) [1]. Under conditions of high energy demand, such as during exercise, AMPK is activated both via AMP level consequent to ATP hydrolysis and through phosphorylation, triggering cellular catabolic pathways and mitochondrial biogenesis while inhibiting anabolic processes. This signaling enhances ATP availability, which is essential for sustaining the mechanical work of muscle fibers [1].

The altered mechano‐metabolic coupling is a hallmark of muscular dystrophies, for example, Duchenne muscular dystrophy (DMD). DMD is a rare and degenerative neuromuscular disorder with an X‐linked recessive inheritance pattern [2]. It is caused by mutations in the gene encoding dystrophin, a subsarcolemmal protein essential for proper mechano‐transduction in muscle fibers. The absence of dystrophin triggers a cascade of pathogenic events, ultimately leading to myofiber necrosis and progressive muscle weakness. Symptoms typically manifest in early childhood, with loss of independent ambulation around 12–13 years of age. In later stages, cardiopulmonary involvement further exacerbates the clinical course, leading to premature death. Currently, no cure exists, and therapeutic approaches (e.g., gold standard corticosteroids) are aimed only to handle symptoms and delay disease progression. In this context, ongoing research is focused on developing new therapies that aim to correct the primary genetic defect or target, at various levels, the cellular pathways dysregulated in the DMD pathogenesis, as among others, metabolism [3], myogenesis [4], inflammation and fibrosis [5], reactive oxygen species (ROS) overproduction [6, 7], and abnormal function of ion channels and other structures involved in altered calcium homeostasis [8].

Dystrophin provides structural support to the myofiber by creating a link between the extracellular matrix and the cytoskeleton, ensuring that mechanical stimuli are effectively translated into biochemical signals that regulate energy metabolism [9]. In this view, it is not surprising that dystrophin absence profoundly disrupts mechano‐metabolic coupling in skeletal muscle. We have previously demonstrated that C57BL/10 *mdx* mice, the mouse model widely used for studying DMD, exhibit insufficient AMPK activation when subjected to a forced standardized chronic exercise protocol [5, 7, 10]. This deficiency blunts the activation of metabolic adaptive signals, leading to a lack of a buffer for the upregulation of pro‐inflammatory and pro‐fibrotic pathways, exacerbating muscle damage and resulting in reduced exercise performance [5, 7, 10]. Conversely, in basal conditions, we observed a chronic activation of AMPK that may have deleterious effects on muscle homeostasis, inhibiting muscle growth and stimulating pro‐atrophic pathways [6, 10, 11]. This pattern was also detected in the D2.B10*‐Dmd* *^mdx^*/ J (D2 *mdx*) mouse model, believed to have a more severe phenotype than that of classic *mdx* mice [11, 12]. AMPK is under the control of two main activators [13]: the calcium/calmodulin‐dependent protein kinase kinase 2 (CAMKK2), which is calcium‐regulated, and the liver kinase B1 (LKB1), regulated by the fluctuation of energy‐related metabolites, such as phospho‐creatine, ATP, and, most importantly, AMP. One possible reason for the chronic activation of AMPK under basal conditions could be the stimulation of CAMKK2 by high intracellular Ca^2^^+^ concentrations that characterize dystrophic myofibers [14]. We have previously demonstrated, for the first time, that the impaired activation of AMPK under exercise stimulation could instead be related to an impairment of the LKB1 pathway [11] and of its interactors.

There has been increasing interest in LKB1, despite being only partially characterized, for its potential crucial role in myofiber homeostasis [15, 16] and in the adaptive response to exercise [17]. It also has an epigenetic function; the kinase, through the phosphorylation‐mediated activation of AMPK and other kinases belonging to the same superfamily, such as salt‐inducible kinases (SIKs) [11, 18], can inhibit the activation of class IIa histone deacetylases (HDAC4 and HDAC5). These deacetylases play a significant role in muscle tissue, acting as epigenetic silencers by repressing the expression of key genes involved in oxidative metabolism and mitochondrial biogenesis, especially in stress conditions [7, 19]. This aspect is particularly relevant considering that HDACs are overactivated in dystrophic muscle [20] and that a pan‐HDAC inhibitor (HDACi), givinostat (Duvyzat, Italfarmaco S.p.A.), has recently been approved by the FDA and EMA for the treatment of DMD [21].

LKB1 is mostly localized at the nuclear level, where it remains inactive. To become active, it must form a complex with two accessory proteins, the pseudokinase STE20‐related adaptor α (STRADα) and the scaffolding mouse protein 25 (MO25; also known as CAB39: calcium binding protein 39), which enable the kinase to migrate to the cytoplasm, where its activity is 10 times higher [22].

In our previous work, we have highlighted that LKB1, STRADα, and MO25 genes and proteins were severely downregulated in gastrocnemius (GC) muscles of both dystrophic mouse models (BL10 *mdx* and D2 *mdx*) in a chronic phase of disease (6–10 months of age) and even more impaired in exercised *mdx* mice [11]. Furthermore, our immunofluorescence (IF) staining showed that *mdx* GC muscle sections had a faint LKB1 signal, mainly present in the nucleus, in contrast with the sarcolemmal localization observed in healthy wild‐type (WT) muscles [11].

Whether these alterations are disease‐specific or rather secondary events due to pathology progression is still unclear. In the present study, we first investigated whether the downregulation of LKB1 is specifically associated with DMD or if it is also observed in other animal models of degenerative disorders characterized by motor impairment (e.g., amyotrophic lateral sclerosis, ALS). Additionally, since our previous research focused solely on an advanced chronic phase of the dystrophic pathology, we aimed to determine whether LKB1 plays an upstream role in the muscle degeneration process observed in dystrophic murine models (both BL10 *mdx* and D2 *mdx*) throughout the lifespan and across three muscles exhibiting distinct profiles: the GC, the diaphragm (DIA), and the heart. Additionally, we investigated the crucial first step underlying the LKB1 dysfunction, focusing on specific microRNAs (miRNAs) likely to be involved in repressing *Lkb1* expression. Although based on limited evidence, miR‐17, miR‐451, and miR‐195 have been identified as potent suppressors of *Lkb1* transcript expression [23, 24, 25] in other pathological settings.

Given the epigenetic role of LKB1, we explored the possibility that its downregulation may be associated with negative feedback regulation by overactive HDACs in murine dystrophic myofibers by means of chronic treatment with two different HDACi. Finally, to enhance the translatability of our study, we also investigated the expression of the LKB1–STRADα–MO25 complex in myoblasts and myotubes derived from DMD pediatric patients.

By undertaking a comprehensive assessment of LKB1 expression and its epigenetic regulation, this study seeks to advance our understanding of the kinase's role in DMD, including uncovering novel mechanistic insights into disease pathogenesis and opening new avenues for the development of targeted therapeutic strategies.

## 2 Materials and Methods

### 2.1 Murine‐Derived Samples: Study Design

All experiments were conducted in compliance with the Italian law for Guidelines for Care and Use of Laboratory Animals (D.L. 116/92), and European Directive (2010/63/UE).

The experiments on ALS murine models, were performed on tibialis anterior (TA) muscles of B6SJL‐TgN (SOD1‐G93A)1Gur mice of 15 weeks of age, expressing high copy number of mutant human *SOD1* coding the Gly93Ala substitution (SOD1G93A) (*n* = 5: 2 males, 3 females), collected for another study (protocol number of Italian Ministry of Health approval: 792/2024‐PR). Muscles of age‐matched B6SJLF1 WT mice were used as control (*n* = 6: 3 males, 3 females). After cervical dislocation, TA muscles were then harvested, snap frozen in liquid N_2_, and stored at −80°C until their utilization for RT‐PCR analyses.

The experiments on dystrophic mouse muscles (GC, diaphragm DIA, and heart) were conducted with male mice belonging to four experimental groups previously used for other studies (protocol number of Italian Ministry of Health approval: DGSAF0024012). Mice were anesthetized via intraperitoneal (i.p.) injection with a cocktail of ketamine (100 mg/kg) and xylazine (16 mg/kg). If required, an additional dose of ketamine alone (30 mg/kg) was injected to ensure longer and deeper sedation. Mice were then sacrificed by cervical dislocation and GC, DIA, and heart muscles were harvested, snap frozen in N_2_, and stored at −80°C until further processing. In detail, samples from the following experimental groups (*n* = 5 each group) were used: male BL10 *mdx* and D2 *mdx* mice of 4, 8, 28, and 52 weeks of age, along with age‐ and sex‐matched WT and DBA 2J (D2 WT) as control. All mice were housed in suitable cages (max. 4–5 mice per cage) filled with sawdust and bedding (LIGNOCEL 3–4S, Charles River Laboratories) and acclimatized to local housing conditions (22°C–24°C, humidity 50%–60%, and 12‐h light/12‐h dark cycle) for approximately 5 days. All mice were fed a daily amount of chow (RM3, Special Diets Services (SDS), Essex, UK) of 5 g per mouse. This fixed amount, largely exceeding the physiological daily consumption (∼3 g/mouse), allowed proper control by the experimenter of any possible macroscopic deviation in food consumption between groups, which in fact did not occur. Filtered tap water was available ad libitum.

For the second part of the study, mice were housed and treated in Italfarmaco's animal facility (Cinisello Balsamo, Milan, Italy), and the experimental protocol was approved by the Italian Ministry of Health (Authorization no. 531/2023‐PR). The two drugs were administered five times a week, intraperitoneally (i.p.) for 8 weeks. Age‐matched D2 *mdx* treated with vehicle (5% DMSO in water) (*n* = 9) and D2 WT also treated with vehicle (5% DMSO in water) (*n* = 9) were used as controls. D2 WT (DBA/2J, stock No: 625) and D2 *mdx* (D2.B10‐*Dmd* *^mdx^*/J, stock No: 013141) 5/6 weeks' old male mice were purchased from Charles River (France) and The Jackson Laboratories (Bar Harbor, Maine, USA), respectively. In each cage, four to five mice were housed (surface area: 800 cm^2^ − BLUE LINE NEXT × IVCs FOR RATS − 1291H—Tecniplast). Mice were kept under pathogen‐free conditions with a 12‐h light/12‐h dark cycle at a temperature of 22° ± 2° and 55% ± 10% humidity. All mice were maintained under a controlled diet (VRF1 diet, Charles River), with a daily amount of chow of 4–5 g/mouse throughout the experiment, and received water ad libitum. Each cage was enriched with a mouse house and nesting material. Mice were regularly checked by a certified veterinarian who was responsible for health monitoring, animal welfare supervision, experimental protocols, and procedure revision. At the end of the treatment, each mouse was anesthetized with isoflurane (4%) and then sacrificed by cervical dislocation. GC muscle was frozen in liquid nitrogen (N_2_) and stored at −80° until being further processed for real‐time PCR and western blot analysis.

### 2.2 In Vivo Functional Test

Run‐to‐exhaustion performance of D2 *mdx* mice was evaluated at 0, 3, 6, and 8 weeks of treatment using a treadmill apparatus (Ugo Basile SRL, Italy) at Italfarmaco laboratories. The exhaustion protocol consisted of an initial horizontal run at 5 m/min for 5 min, after which the speed was increased by 1 m/min every minute until exhaustion (the procedure was compliant with the standard operating procedures of TREAT–NMD Neuromuscular Network (SOP ID DMD_M.2.1.003) [26]). The test was concluded when mice remained for more than 10 s on the shocker plate. Mice were randomized into homogeneous groups based on body weight and baseline running performance. Treadmill tests were conducted after a training period of 4 days and after an acclimatization period during which mice became familiar with the procedure. Run to exhaustion test was performed during the light‐cycle phase, in the early hours of the morning (8:00–12:00 am).

### 2.3 Creatine Kinase (CK) Measurements

In Italfarmaco, before being sacrificed by cervical dislocation, blood samples were collected from the retro‐orbital plexus of D2 mice, transferred into tubes containing heparin as anticoagulant (100 USP units/mL), and then centrifuged for 10 min at 13,000 rpm at 4°C. A second centrifugation step (10 min at 12,000 rpm) was performed to ensure complete platelet removal; then, samples were stored at −80°C until use.

CK levels (in U/L) were measured in plasma samples by means of commercially available diagnostic kits (CK NAC LR, SGM, Rome, Italy) [27]. The assay required the use of a spectrophotometer (Ultrospec 2100 Pro UV/Visible, Amersham Biosciences, Little Chalfont, UK) set to a wavelength of 340 nm at 37°C, and was performed according to the manufacturer's instructions.

### 2.4 Cell Cultures

We used human‐derived immortalized cell lines, provided from the biobank MyoLine, previously immortalized by the Vincent Mouly lab: HWT (AB1190) and HDMD (deletion 48–50; AB1098). Cells were cultured in flasks and maintained in a proliferative state (myoblast) at 37°C using a proliferation medium made of basal medium (skeletal muscle cell growth medium; PromoCell C‐23260), 15% fetal bovine serum (FBS; Gibco 10270106), 1% gentamicin (Gibco 15750037), 1% glutaMax (Gibco 35050038), 5% growth medium supplement mix (PromoCell C‐39365). Differentiation was initiated by using a differentiation basal medium (skeletal muscle differentiation medium; PromoCell C‐23061) with the addition of 2% differentiation medium supplemental mix (PromoCell C‐23061) and 2% FBS (Gibco) [28].

### 2.5 Isolation of Total RNA, Reverse Transcription, and RT‐PCR

Total RNA was isolated from muscle cryosections (GC, DIA, TA, and heart) obtained using a cryostat (100–150 sections, 10 µm thick; approximately 150–200 mg of tissue). Sections were collected into two prechilled microcentrifuge tubes (one for RT‐PCR and one for western blot analyses) and stored at −80°C until use. Room temperature (RT) lysis buffer (200 µL) from the miRVana kit (cat. No. A27828, Thermo Fisher Scientific, Waltham, MA, USA) containing β‐mercaptoethanol (1.4 µL) was then added directly to the samples. Solution and tissue were homogenized by pipetting to ensure proper lysis.  Then, the extraction was performed by the KingFisher Duo Prime system (Thermo Fisher Scientific). RNA purity was evaluated via nanodrop spectroscopy (ND‐1000 NanoDrop, Thermo Fisher Scientific) to confirm 260/280 ratios were >2.0, and 260/230 ratios >1.8.

About 400 ng of purified RNA were reverse transcribed to cDNA with iScript gDNA Clear cDNA Synthesis Kit (172–5035 Bio‐Rad Laboratories Inc., CA, USA) [27]. To each RNA sample, 2 µL of DNase master mix (volume by reaction: iScript DNase 0.5 µL, iScript DNase Buffer 1.5 µL) and nuclease‐free water for a total reaction volume of 16 µL were added. The reaction was incubated using a thermal cycler at conditions 25° for 5 min, followed by 75° for 5 min to inactivate DNase. Four microliters of iScript Reverse Transcription Supermix 5× was then added, with the reaction incubated at 25° for 5 min, followed by 46° for 20 min with a final heat inactivation of 95° for 1 min, according to the protocol provided by the manufacturer. Eighty microliters of RNAse‐free water was then added to the sample in order to obtain a 4 ng/µL cDNA solution. Quantitative real‐time (RT) PCR was performed via the CFX384 Connect Real‐Time PCR system (Bio‐Rad, Hercules, CA, USA) by using Prime PCR assays SYBR green. Each reaction used 2 µL of cDNA (8 ng cDNA, assuming 1:1 conversion), added with 5 µL of SssoAdvanced Universal SYBR Green Supermix (172–5271 Bio‐Rad Laboratories Inc., CA, USA), 0.5 µL of primers, and 2.5 µL of nuclease‐free water for a total volume of 10 µL. All RT‐PCR performed including SYBR Green used a hot start (95°C for 2 min) followed by 40 cycles of a two‐step reaction (95°C for 5 s, 60°C for 30 s). For each experiment, samples were analyzed in technical triplicate. The raw data were preliminarily screened and only technical triplicates with a standard deviation <0.8 were considered acceptable for the analysis. All primer sequences for candidate reference genes are proprietary property of Bio‐Rad Laboratories: *Lkb1* (Assay ID qMmuC ID0006462), *Strada* (Assay ID qMmuC ID0014831), *Mo25* (*CAB39*, Assay ID qMmuC ID0022694), myocyte enhancer factor 2C (*Mef2c*, Assay ID qMmuCID0005168), peroxisome proliferator‐activated receptor gamma coactivator 1 alpha, (*Pgc1a*, Assay ID qMmuCID0006032), and autophagy related 12 (*Atg12*, qMmuCID0016287). For GC and DIA muscles, the average mRNA expression of target genes was normalized to the mean of two housekeeping genes [29], casein kinase 2 alpha 2 (*Csnk2a2*, Assay ID qMmuC ID0026331) and adaptor‐related protein complex 3 subunit delta 1 (*Ap3d11*, Assay ID qMmuC ID0022103), and quantified by the 2^ΔΔct^ method [30]. For the heart, data were normalized to the mean of the HIV‐1 tat specific factor 1 gene (*Htatsf1*, Assay ID qMmuCID0013600) and the succinate dehydrogenase complex flavoprotein subunit A gene (*Sdha*, Assay ID qMmuCID0019353). To assess gene expression in human cells, we use the following Bio‐Rad gene primers: *LKB1* (Assay ID qHsaCID0008647), *STRADA* (Assay ID qHsaCID0016267), *MO25* (*CAB39*, Assay ID qHsaCID0006255), myosin heavy chain 2 (*MYH2*, Assay ID qHsaCID0012805), and myogenin (*MYOG*, qHsaCED0022985). Data were normalized to the mean of the ribosomal protein S18 gene (*RPS18*, Assay ID qHsaCED0037454) and the ubiquitin C gene (*UBC*, qHsaCED0023867) [31]. For miRNA analysis, first, we performed a reverse transcription using the TaqMan miRNA Reverse Transcription Kit (cod. 10268364, Thermo Fisher Scientific) following the manufacturer's instructions. qRT‐PCR was then performed using the CFX384 Connect Real‐Time PCR System (Bio‐Rad, Hercules, CA, USA) and the TaqMan TM Small RNA Assay (cod. 4398987, Thermo Fisher Scientific). For each experiment, samples were analyzed in technical triplicate. The expression of miRNA was normalized to the housekeeping gene U6 snRNA and quantified by the 2^ΔΔct^ method [30]. Probes were purchased from Thermo Fisher Scientific with the following Unique Assay IDs: miR‐451‐cp Assay ID: rno481395_mir; miR‐195‐5p, Assay ID: rno480882_mir; has‐mir‐17‐5p, Assay ID:478447_mir; U6 snRNA, Assay ID:001973.

### 2.6 Protein Expression Analysis by Western Blot

Protein extractions and immunoblots for the determination of targets of interest were performed in left GC muscles of D2 *mdx* mice treated with vorinostat and Rodin‐A. GC muscles of untreated D2 *mdx* and D2 WT mice were used as controls. Cryosections of all muscles were obtained as described in paragraph 2.5, and homogenized in ice‐cold buffer containing 20 mM Tris–HCl (pH 7.4 at 4°C), 2% SDS, 5 mM EDTA, 5 mM EGTA, 1 mM DTT, 100 mM NaF, 2 mM sodium vanadate, 0.5 mM phenylmethylsulfonyl fluoride, 10 mg/mL leupeptin, and 10 mL/mL pepstatin. Homogenates were centrifuged at 1500 *g* for 10 min at 4°C, and the supernatant was quantified using the BCA Protein Assay Kit (Sigma‐Aldrich, Saint Louis, MO, USA) according to manufacturer's instructions. For immunoblot analysis, 40 µg of protein were separated on a 10% SDS‐PAGE and transferred onto PVDF membrane for 7 min at 1.3A–25 V (Trans‐Blot Turbo Transfer System, Bio‐Rad Laboratories Inc). The following dilutions of primary antibodies were used: 1:1000 anti‐vinculin (7F9 sc‐73614 Santa Cruz Biotechnology); 1:700 rabbit anti‐LKB1 (Cell Signaling Technology Inc., MA, USA, BK3047S); 1:700 rabbit anti‐MO25α/CAB39 (Cell Signaling Technology Inc., BK2716S); 1:300 rabbit anti LYK5 (STRADα) (Sigma‐Aldrich, AV49077). For the determination of glucose transporter type 4 (GLUT4) (1:700, sc‐53566, Santa Cruz Biotechnology) and salt inducible kinase 1 (SIK1) immunoprecipitation was performed prior to immunoblot analysis as previously described [26, 32]. The depleted lysate of the immunoprecipitated samples was used for the detection of vinculin. For SIK1, the immunoprecipitated was divided into two aliquots: one was resolved by SDS‐PAGE and probed with anti‐SIK1 antibody (1:400, PA5‐42799, Thermo Fisher Scientific Inc) to detect total immunoprecipitated SIK1, and the other was probed with anti‐phospho‐SIK1 (1:400, Thr182, PA5‐105918, Thermo Fisher Scientific Inc) to detect the phosphorylated form.

For the determination of LKB1 in HWT and HDMD cells at 11 days of differentiation, no fewer than one million cells were used in the experiment. Cells were harvested in 200 µL of cold RIPA buffer (20 mM Tris–HCl, 150 mM NaCl, 1.5% Nonidet P‐40, 100 mM sodium orthovanadate, and a protease inhibitor cocktail) and placed on ice for 10 min. Cell lysates were centrifuged at 1500 *g* for 10 min at 4°C and the supernatant was quantified using the BCA Protein Assay Kit. For immunoblot analysis, 60 µg of protein were separated on a 10% SDS‐PAGE and then transferred onto PVDF membrane for 7 min at 1.3A–25 V (Trans‐Blot Turbo Transfer System, Bio‐Rad Laboratories Inc). The following dilutions of primary antibodies were used: 1:100 human primary anti‐LKB1 (sc‐374334 Santa Cruz Biotechnology) and 1:1000 anti‐vinculin (7F9 sc‐73614 Santa Cruz Biotechnology).

Anti‐mouse (1:5000 anti‐mouse IgG, Sigma‐Aldrich) and anti‐rabbit (1:5000 anti‐mouse IgG, Bio‐Rad Laboratories) horseradish peroxidase‐conjugated secondary antibodies were used. Densitometric analysis was performed using image laboratory software (Bio‐Rad Laboratories). LKB1, MO25α, STRADα, and GLUT4 levels were normalized to vinculin. p‐SIK1 levels were normalized to total immunoprecipitated SIK1 to determine the relative phosphorylation state of the protein. (The software allows the chemiluminescence detection of each experimental protein band to obtain absolute signal intensity.) The density volume was automatically adjusted for by subtracting the local background [26, 33, 34].

### 2.7 Immunofluorescence Analysis

HWT and HDMD cells were grown on IBIDI µ‐Slide (Cod. 80807‐120, IBIDI GmbH) at a density of 3 × 10^4^ cells for 11 days in technical triplicate. Cells were fixed in 4% paraformaldehyde for 10 min at RT and, after three washes of 5 min in PBS, were then permeabilized with 0.1% TWEEN 20 (Sigma‐Aldrich) and 2% bovine serum albumin (Sigma‐Aldrich) in PBS for 45 min at RT. Cells were blocked in saturation buffer (2% bovine serum albumin in PBS) and incubated with human monoclonal anti‐LKB1 primary antibody (1:50; sc‐374334, Santa Cruz Biotechnology) for 1 h at RT in blocking buffer. After three washes in PBS, cells were incubated with 594 Alexa Fluor‐conjugated secondary antibodies (1:1000; A32744, Thermo Fisher Scientific) for 45 min at RT. After three washes of 5 min with PBS, nuclei were stained with PureBlu Hoechst 33342 Nuclear Staining Dye (1:100; Bio‐Rad). Phalloidin‐Atto 488 (1:400—Invitrogen A12379) was used to mark the cytoskeleton (green signal). Images were acquired using a Leica THUNDER Imager (11525687, Leica Microsystem) and Las X Inc. software (Leica Microsystem) with a 20× objective. Fluorescence quantification was performed using CellProfiler 4.x (Broad Institute). Composite RGB images were split into individual channels using the ColorToGray module. Nuclei were segmented on the Hoechst channel (blue) using the IdentifyPrimaryObjects module, and cell boundaries were defined on the GFP channel (phalloidin, green) using the “IdentifySecondaryObjects” module with a propagation method, using nuclei as seeds. Integrated fluorescence intensity of the red channel was measured for each individual cell using the “MeasureObjectIntensity” module and exported as numerical data. All images were acquired in a single microscopy session under identical conditions, ensuring comparability across samples.

To analyze myotube formation, we performed immunostaining for F‐actin, myosin II—heavy chain, and nuclei. Around 250k cells/well were cultured in a six‐well plate (CytoOne—Starlab) for 6 and 11 days in differentiation medium. At each time point, cells were fixed in 4% paraformaldehyde in PBS for 10 min at RT and then washed three times with PBS and then blocked in blocking buffer (2% BSA + 1% Triton X‐100 in PBS) for 45 min. Anti‐myosin primary antibody (MF‐20‐S; 1:10; Developmental Studies Hybridoma Bank) was diluted in blocking buffer (w/Triton) and incubated at RT for 1 h. After three washes in PBS, cells were incubated with 594 Alexa Fluor‐conjugated secondary antibodies (1:500; Thermo Fisher Scientific) diluted in blocking buffer (w/ Triton) for 45 min at RT. Nuclei were stained with Pure Blu Hoechst nuclear staining dye (1:100; Bio‐Rad). Mounting medium was added before imaging. Images were acquired using a Leica THUNDER Imager (11525687) with a 20× objective. To assess the nuclear fusion index, random regions of interest (ROIs) were acquired per well. Images were analyzed using Fiji ImageJ software. The experiment was replicated three times with different cell vials (*N* = 3). The number of nuclei within myotubes was counted using a multipoint tool, considering cells as multinucleated if they present >2 nuclei and are positive for MF‐20 staining. Fusion index for each ROI was then calculated with the following formula: (number of nuclei in multinucleated cells/number of total nuclei present in a given field) × 100.

In vitro imaging for mitochondria was performed on human cell lines seeded in differentiation medium in a black 96‐well plate with optical filter (uClear GREINER 655090, Sigma‐Aldrich, USA) at a density of 30 × 10^3^ cells per well, in technical quadruplicate. After 11 days of differentiation, the staining was performed. Following 15 min of RT exposure, cells underwent mitochondrial staining with MitoTracker Orange CMTMRos dye (Thermo Fisher Scientific), along with nuclear staining (PureBlu Hoechst 33342, Bio‐Rad, USA; 1:100). Then, cells were fixed with paraformaldehyde (PFA 2%v/v) for 30 min at room RT and washed with PBS. The experiment was replicated three times with different cell vials (*N* = 3). To evaluate the content of active mitochondria, random regions per well were analyzed using the Operetta CLS system (PerkinElmer, USA) (20× magnification) and Harmony software (v 3.5, PerkinElmer). For each well, MitoTracker fluorescence intensity was quantified and normalized by the total number of nuclei.

### 2.8 Statistical Analysis

Data are expressed as mean ± SEM. The Shapiro–Wilk test was used to assess the normal distribution of data and the possibility to apply statistical parametric tests [35]. In this case, the unpaired Student's *t*‐test was used when comparing two independent experimental conditions. In relation to the data concerning pharmacological treatment, a single comparison between means by the unpaired Student's *t*‐test was only used to address differences between WT and dystrophic mice. Multiple statistical comparisons among dystrophic mice groups (untreated D2 *mdx*, D2 *mdx* + vorinostat, D2 *mdx* + Rodin‐A) were performed by a one‐way ANOVA with Dunnett's test post hoc correction. Statistical analyses were performed using GraphPad Prism version 8. Only for the analysis of cardiac *Lkb1* expression at the 52‐week time point, a two‐way ANOVA was performed to simultaneously evaluate all four experimental groups. The null hypothesis was rejected at *p* < 0.05 for all statistical analyses.

Whenever appropriate, the recovery score (RS), an objective index directly indicating how much of the deficit is recovered (%) by a treatment, was calculated according to TREAT‐NMD (SOP (ID) Number: DMD_M.1.1.001), as follows:

$$\mathrm{Recovery}\;\mathrm{score}=\frac{\left(\mathrm{treated}\;mdx\;\mathrm{mice}-\mathrm{untreated}\;mdx\;\mathrm{mice}\right)}{\left(\mathrm{control}\;\mathrm{mice}-\mathrm{untreated}\;mdx\;\mathrm{mice}\right)}$$

## 3 Results

### 3.1 *Lkb1–Strada–Mo25* Complex Was Not Dysregulated in a Murine Model of ALS

**FIGURE 1:**
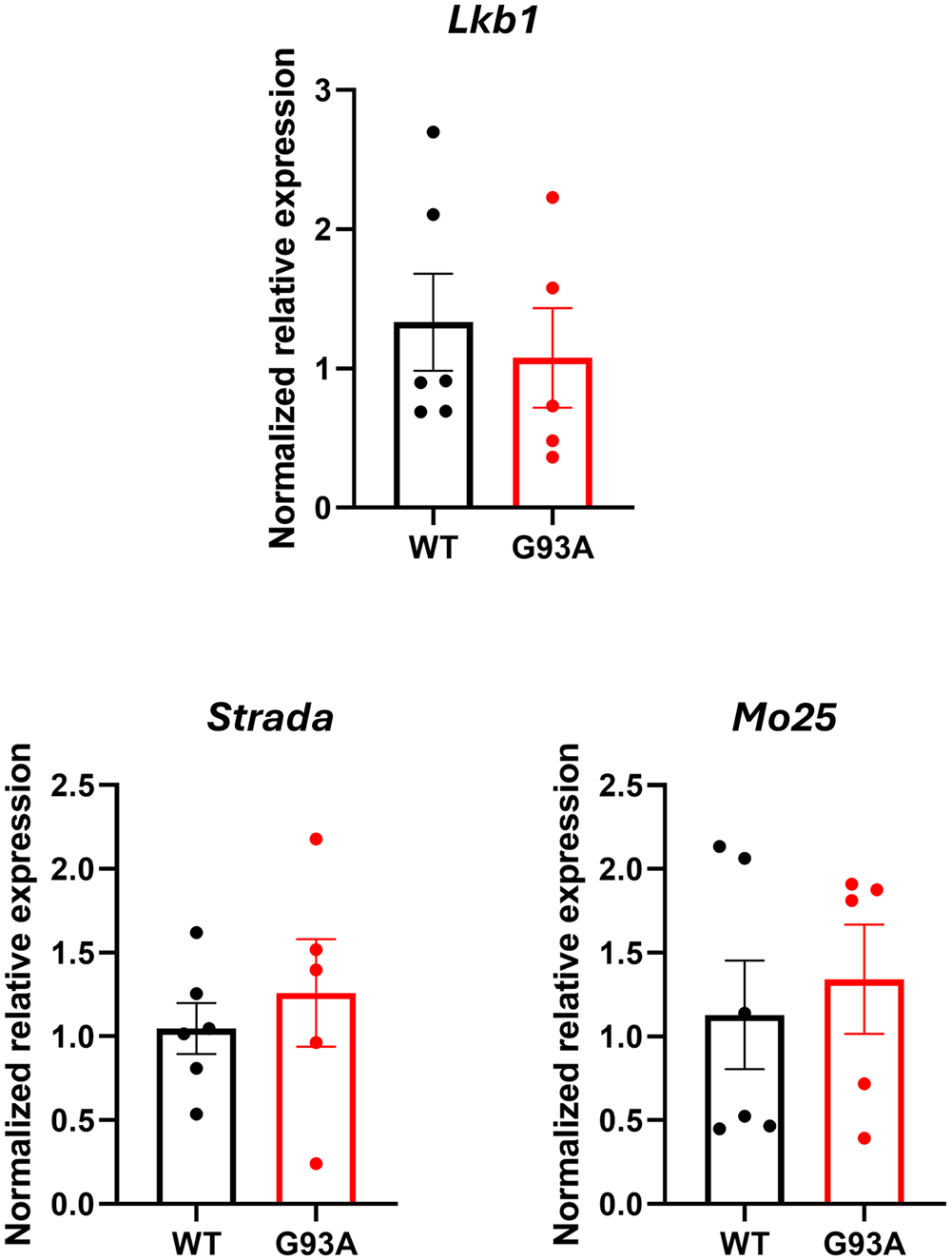
*Lkb1–Strada–Mo25* gene expression in tibialis anterior muscles of an amyotrophic lateral sclerosis mouse model (SOD1‐G93A). Data are expressed as mean ± SEM (*n* = 5–6). No significant differences between samples were found (unpaired Student's *t*‐test).

RT‐PCR analysis in TA muscles of SOD1 G93A mice showed no difference in *Lkb1–Strada–Mo25* expression in comparison with WT mice (Figure 1). However, the mice exhibited all the pathological features typical of the model, for example, limb paralysis, reduced locomotion, and neuroinflammation (data not shown).

### 3.2 Dystrophic Mice Showed an Impairment of *Lkb1–Strada–Mo25* Expression throughout Lifespan

**FIGURE 2:**
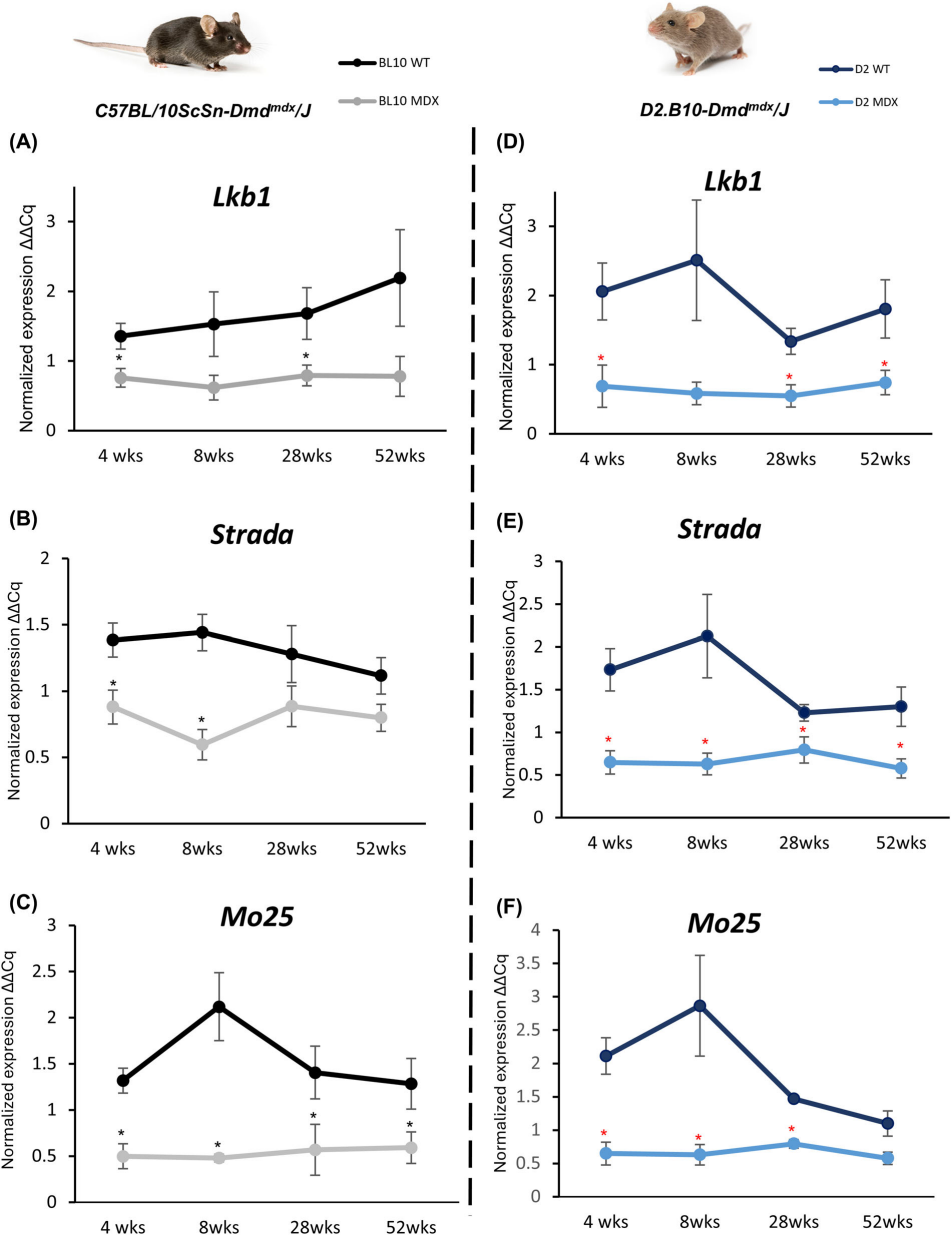
*Lkb1–Strada–Mo25* gene expression in gastrocnemius muscles of two Duchenne muscular dystrophy mouse models throughout lifespan. Data are expressed as mean ± SEM. Significant differences were assessed by an unpaired Student's *t*‐test. *Lkb1*: (A) BL10 *mdx* mice (*n* = 4–5) versus BL10 WT mice (*n* = 4–5) (*0.042 *< p <* 0.045); (D) D2 *mdx* mice (*n* = 4–5) versus D2 WT mice (*n* = 4–5) (*0.01 < *p* < 0.048). *Strada*: (B) BL10 *mdx* mice (*n* = 4–5) versus BL10 WT mice (*n* = 4–5) (*0.001 *< p <* 0.023); (E) D2 *mdx* mice (*n* = 4–5) versus D2 WT mice (*n* = 4–5) (*0.005 *< p <* 0.04). *Mo25*: (C) BL10 *mdx* mice (*n* = 4–5) versus BL10 WT mice (*n* = 4–5) (*0.001 *< p <* 0.04); (F) D2 *mdx* mice (*n* = 4–5) versus D2 WT mice (*n* = 4–5) (*0.0001 < *p* < 0.02).

The results of RT‐PCR analysis of *Lkb1– Strada–Mo25* genes on GC muscles of all experimental groups are shown in Figure 2. The expression of all three genes was severely and persistently downregulated in both dystrophic mouse models, at all ages, with an overall reduction of more than 50% versus background‐related WT mice. Notably, this evidence was robust across all tested conditions. In fact, confirming the data previously obtained during the chronic stage of the disease (6–10 months), our results also showed that the impairment in the complex expression, and particularly of *Lkb1*, was also statistically significant during the acute phase (4–8 weeks of age) and persisted into the late stage (52 weeks, Student's *t*‐test: Figure 2A (0.042 < *p* < 0.045; 2D 0.01 < *p* < 0.048).

**FIGURE 3:**
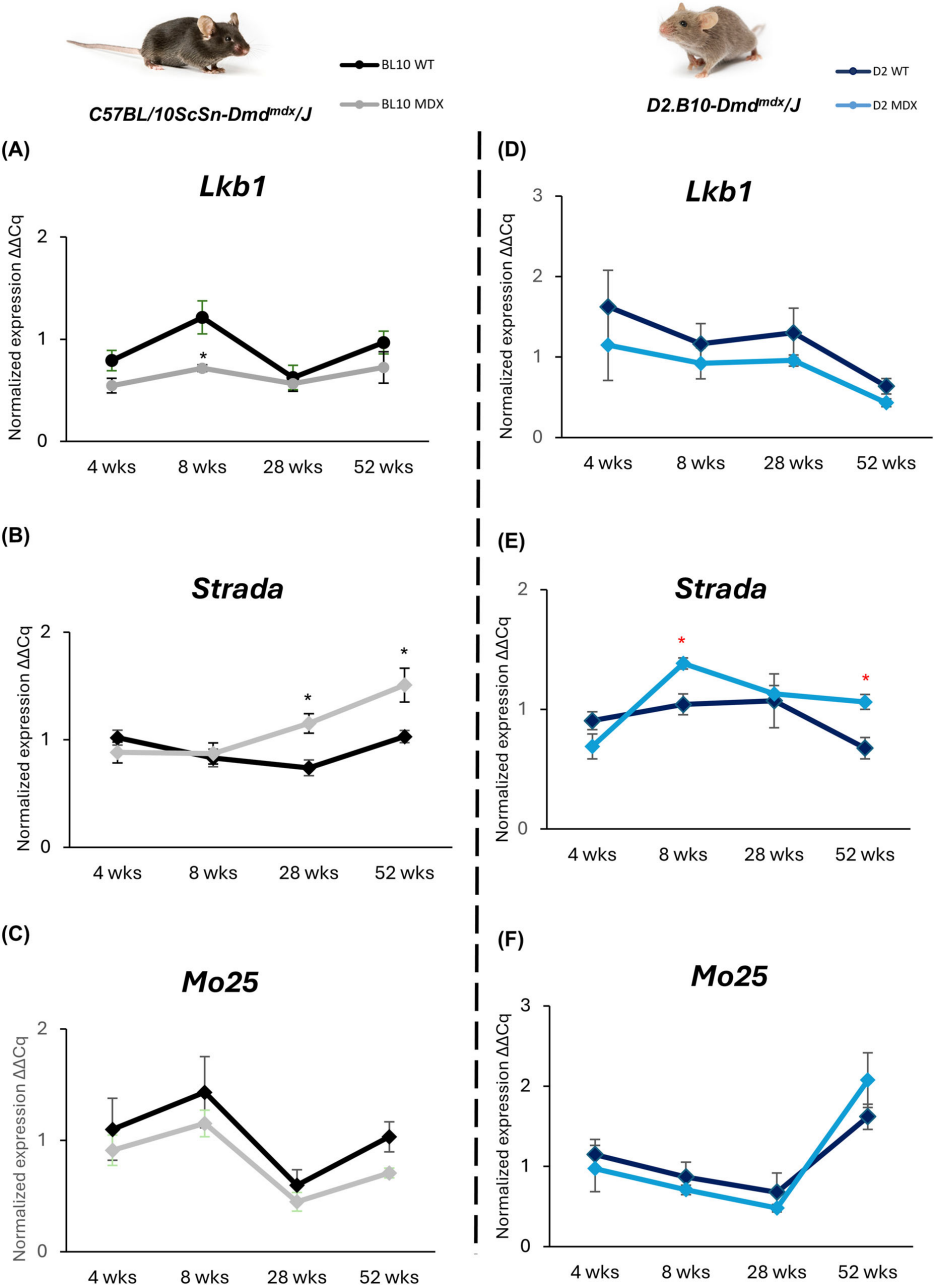
*Lkb1–Strada–Mo25* gene expression in the diaphragm muscles of two Duchenne muscular dystrophy mice throughout lifespan. Data are expressed as mean ± SEM. Significant differences were assessed by an unpaired Student's *t*‐test. *Lkb1*: (A) C57BL/10 *mdx* mice (*n* = 4–5) versus C57BL/10 WT mice (*n* = 4–5) (*p = 0.017). *Strada*::(B) C57BL/10 *mdx* mice (*n* = 4–5) versus C57BL/10 WT mice (*n* = 4–5) (*0.007 < *p* < 0.03); (E) D2 *mdx* mice (*n* = 4–5) versus D2 WT mice (*n* = 4–5) (*0.007 < *p* < 0.008). *Mo25*: No significant differences were observed.

We conducted the same set of experiments in DIA (Figure 3), as this muscle is severely affected in DMD. Our results showed that *Lkb1* (and similarly *Mo25*) expression was not significantly affected in either dystrophic model at any age examined, with only a mild trend toward reduction. In contrast, the expression levels of *Strada* were significantly higher in both dystrophic models from 8 weeks onwards (Figure 3B, Student's *t*‐test 0.007 < *p* < 0.03; 3E: 0.007 < *p* < 0.008).

Given that hypertrophic cardiomyopathy has been reported in DMD patients as well as in dystrophic mouse models at advanced stages of the disease and represents the leading cause of premature death in these patients, we also performed RT‐PCR analyses in the heart muscles of both dystrophic models at 8, 28, and 52 weeks of age. An earlier time point (4 weeks) was not investigated, as it is well established that no overt cardiac pathology is present at this stage of disease in animal models, since cardiac involvement typically manifests around 10 months of age in *mdx* mice and at 6–8 months of age in D2 *mdx* mice [12].

**FIGURE 4:**
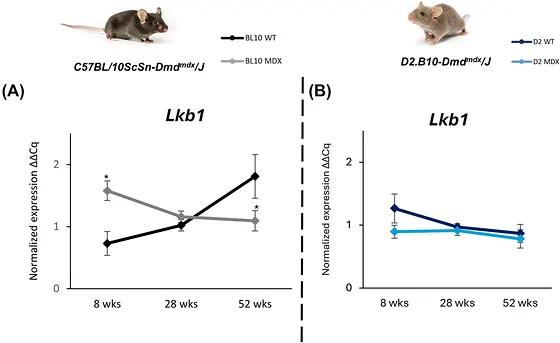
*Lkb1* gene expression in heart muscles of two Duchenne muscular dystrophy mouse models at 8–28–52 weeks of age. Data are expressed as mean ± SEM. Significant differences were assessed by an unpaired Student's *t*‐test. (A) BL10 *mdx* mice (*n* = 4–5) versus BL10 WT mice (*n* = 4–5) (*0.009 < *p* < 0.01). (B) No significant differences were observed.

The two dystrophic models display distinct temporal patterns in *Lkb1* expression (Figure 4). In the classical *mdx* model, there is a progressive decline in the kinase gene expression over time, whereas the D2 *mdx* mice consistently show stable expression levels across all ages examined. Notably, *Lkb1* expression was significantly higher in classical *mdx* mice compared to BL10 WT mice at 8 weeks of age and markedly decreased at 52 weeks, an age at which cardiac pathology is fully established (Student's *t*‐test: 0.009 < *p* < 0.01). A decrease of *Lkb1* expression was also observed over time in the D2 WT mice, a model that is not exempt from developing signs of cardiac pathology (e.g., the appearance of calcifications) [12]. Taken together, analysis of gene expression across the four experimental groups revealed that the D2 genotype consistently displayed lower expression levels than the BL10 genotype, especially at the late time point (normalized relative expression at 52 weeks with inter‐run calibration: BL10 WT 1.8 ± 0.35; BL10 *mdx* 1.09 ± 0.16; D2 WT 0.86 ± 0.14; D2 *mdx* 0.78 ± 0.14). Two‐way ANOVA analysis revealed a significant main effect of strain on *Lkb1* expression at 52 weeks (F (1,16) = 22.04, *p* = 0.0002), indicating that the D2 strain displays lower baseline *Lkb1* expression than BL10.

### 3.3 *Lkb1‐Strada‐Mo25* Expression Was Upregulated by HDAC Inhibitors

**FIGURE 5:**
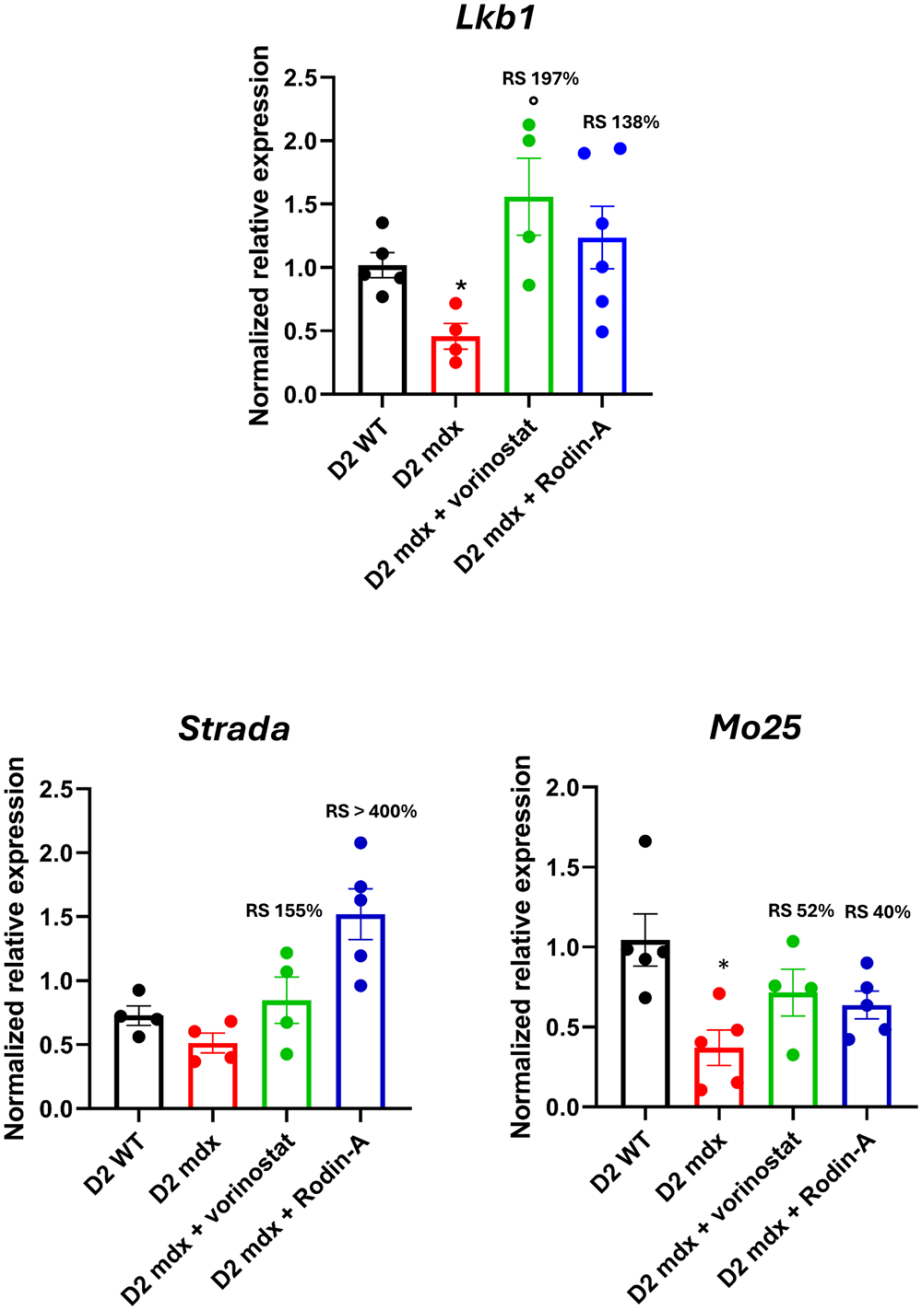
*Lkb1–Strada–Mo25* gene expression in gastrocnemius muscles of D2 *mdx* mice treated with HDAC inhibitors. All values are expressed as mean ± SEM. For *Lkb1* and *Mo25*, a statistically significant difference was found via an unpaired Student's *t*‐test for D2 *mdx* versus D2 WT mice (*0.003 < *p* < 0.006). For *Lkb1*, a statistically significant difference was found among D2 *mdx* mice via a one‐way ANOVA (F (3,20) = 4.7, *p* = 0.03). Dunnett's post hoc test, used to compare each treated group to the vehicle group, is as follows: ° versus D2 *mdx* (*p* < 0.02).

We conducted RT‐PCR analyses to evaluate the expression of the *Lkb1‐Strada‐Mo25* axis in the GC muscles of 5‐week‐old D2 *mdx* mice treated for 8 weeks with either the broad‐spectrum HDACi vorinostat or the COREST complex selective HDAC1/2 inhibitor Rodin‐A. As shown in Figure 5, the expression of all three genes was markedly reduced in D2 *mdx* mice compared to D2 WT controls (Student's *t*‐test 0.003 < *p* < 0.006), even at this age (≈ 13 weeks of age). Notably, our results reveal a previously unrecognized mechanism of HDACi: both treatments, in fact, significantly increased the gene expression levels of the heterotrimeric complex. Vorinostat robustly restored the expression of all target genes, with *Lkb1* showing a particularly notable and statistically significant recovery (RS 197%, one‐way ANOVA F (3,20) = 4.7, *p* = 0.03; Dunnett's post hoc test, vs. D2 mdx *p* < 0.02).

Rodin‐A similarly enhanced *Lkb1* expression (RS 138%), although its effect was more pronounced on the upregulation of *Strada* (> 400%). More modest effects were observed on *Mo25* expression following both treatments (vorinostat RS = 52%; Rodin‐A RS = 40%).

**FIGURE 6:**
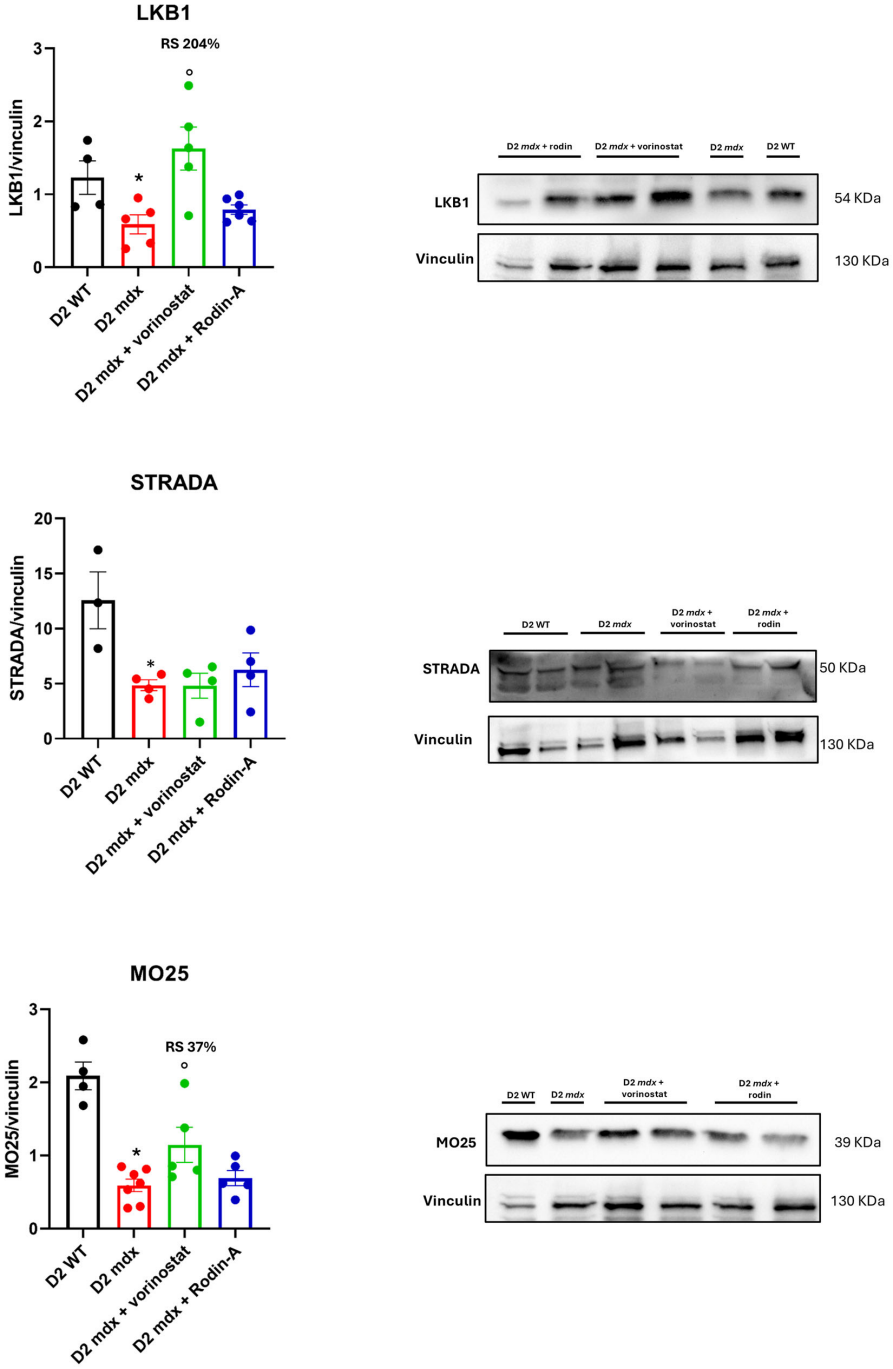
LKB1–STRADα–MO25 protein expression in gastrocnemius muscles of D2 *mdx* mice treated with HDAC inhibitors. Blots were loaded by an operator unaware of the experimental groupings of samples. All values are expressed as mean ± SEM. for the number of mice. For LKB1, STRADα, and MO25, a statistically significant difference was found via an unpaired Student's *t*‐test for D2 *mdx* versus D2 WT mice (*0.0001 < *p* < 0.02). For LKB1 and MO25, a statistically significant difference was found among D2 *mdx* mice via a one‐way ANOVA (LKB1: F(2,13) = 3.8, *p* < 0.05, MO25: F(2,15) = 3.9, *p* < 0.04). Dunnett's post hoc test, used to compare each treated group to the vehicle group, is as follows: ° versus D2 *mdx* (0.03 < *p* < 0.04).

Western blot analysis (Figure 6) partially corroborated these transcriptional findings at the protein level. In vorinostat‐treated muscles, LKB1 protein levels significantly increased (RS + 204%, one‐way ANOVA F *=* 3.8, *p* < 0.05, Dunnett's post hoc test vs. D2 *mdx p =* 0.03), while MO25 displayed a more modest result (RS 37%, one‐way ANOVA F *= 3.9, p <* 0.04, Dunnett's post hoc test vs. D2 *mdx p* = 0.04). No significant changes were detected for STRADα with either treatment, nor for LKB1 or MO25 in response to Rodin‐A.

### 3.4 *Lkb1*‐Silencing miRNAs Were Downregulated by the HDAC Inhibitor Vorinostat

**FIGURE 7:**
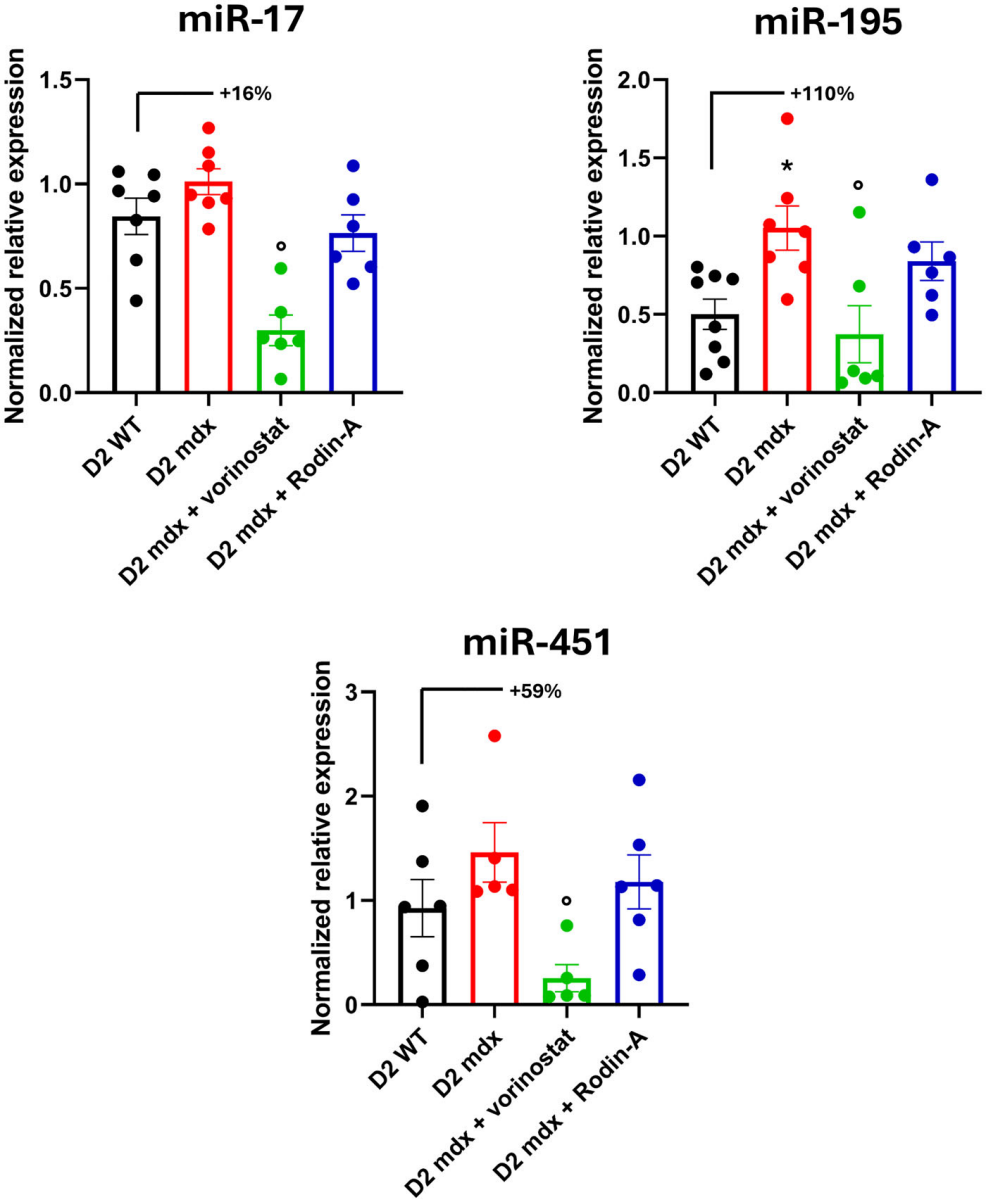
miRNA expression in gastrocnemius muscles of D2 *mdx* mice treated with HDAC inhibitors. All values are expressed as mean ± SEM. For miR‐195, a statistically significant difference was found via an unpaired Student's *t*‐test for D2 *mdx* versus D2 WT mice (*p < 0.02). For all three miRNAs, a statistically significant difference was found among D2 *mdx* mice via one‐way ANOVA: miR‐17: F (2,16) = 24.27, *p* < 0.0001; miR‐195: F (2,16) = 5.37, *p* < 0.016; miR‐451: F (2,16) = 6.74, *p* < 0.007). Dunnett's post hoc test, used to compare each treated group to the vehicle group, is as follows: ° versus D2 *mdx* (0.0001 < *p* < 0.01). The percentage increase in the expression of miR‐17, ‐195, and ‐451 in D2 *mdx* mice compared to D2 WT (+16%, +110%, and +59%, respectively) is shown in the graphs.

In the same samples, we analyzed the expression of miRNAs involved in the regulation of *Lkb1* expression (Figure 7). Our data identified a novel critical regulatory node: miR‐195 was significantly upregulated in the muscles of dystrophic mice (Student's *t*‐test, *p* < 0.02), whereas the increase in miR‐451 and miR‐17 expression was less pronounced (+59% and +16%, respectively). Notably, treatment with the pan‐HDAC inhibitor vorinostat significantly reduced the expression of all three miRNAs analyzed (One‐way ANOVA for miR‐17 F(2,16) = 24.27, *p* < 0.0001), miR‐195 F(2,16) = 5.37, *p* < 0.016), and miR‐451 F(2,16) = 6.74, *p* < 0.007). Dunnett's post hoc test vs. D2 *mdx*, 0.0001 < *p* < 0.01), whereas Rodin‐A appeared ineffective in modulating their levels.

### 3.5 LKB1 Restoration Only Partially Translates Into Increased Expression and Activation of Molecular Targets

**FIGURE 8:**
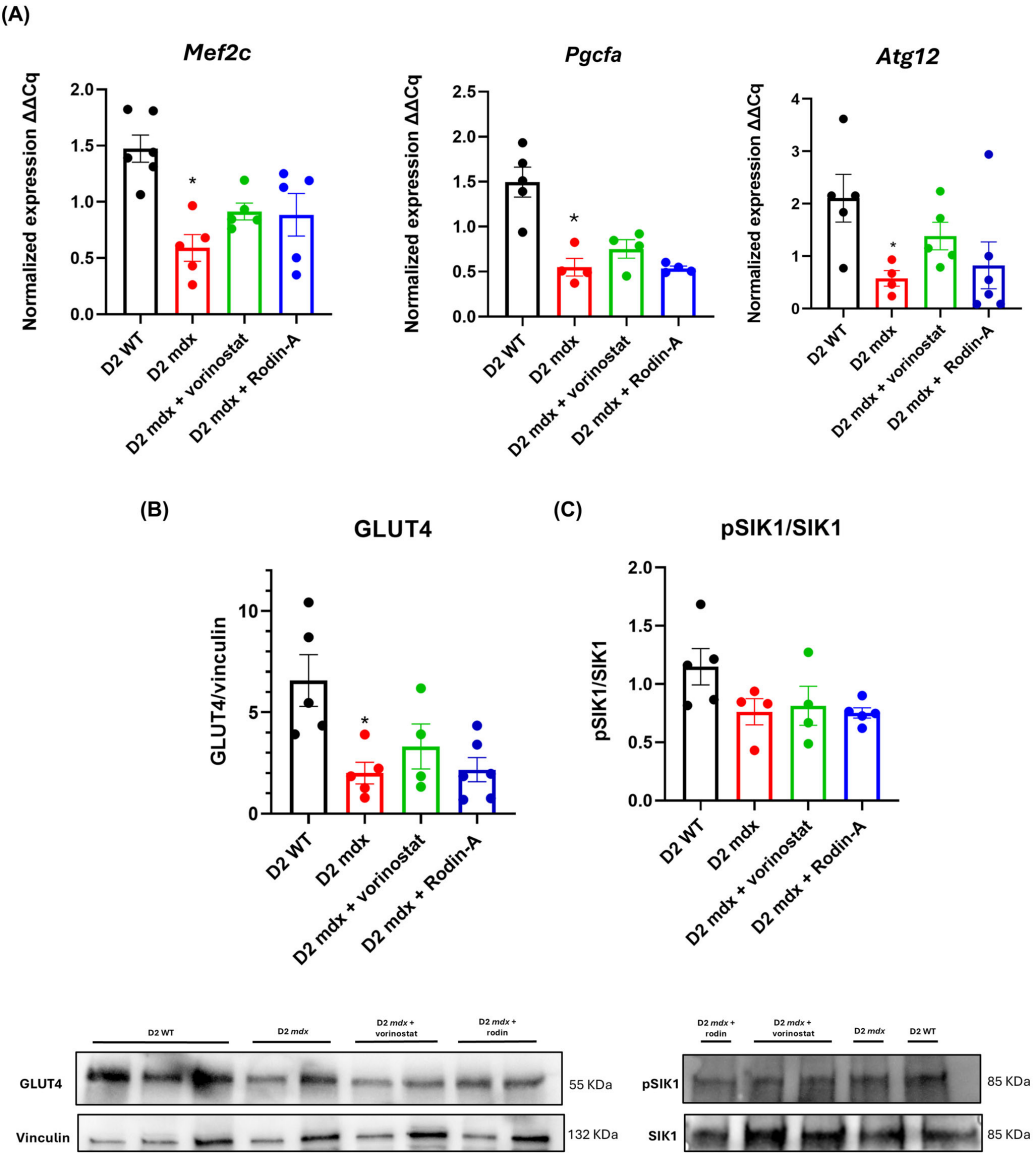
Gene and protein expression of LKB1 targets. All values are expressed as mean ± SEM. For *Mef2c, Pgc1a, Atg12*, and GLUT4, a statistically significant difference was found via an unpaired Student's *t*‐test for D2 *mdx* versus D2 WT mice (*p = 0.006, *p* = 0.0026, *p* = 0.023, and *p* = 0.01, respectively). No significant differences were found for pSIK1/SIK1 ratio. Representative western blot panels displayed below show GLUT4 and pSIK1/SIK1 protein expression. Densitometric quantification of bands is presented in the corresponding graph.

To determine whether the observed restoration of LKB1 is accompanied by the activation of its downstream signaling cascade, we examined key effectors of the LKB1–class IIa HDAC axis (Figure 8). Analysis of the gene expression of *Mef2c* and *Pgc1*α, two transcriptional targets regulated by this axis, revealed a significant reduction in D2 *mdx* mice compared to WT controls (Student's *t*‐test *p* = 0.006 and *p* = 0.0026, respectively). Both targets showed a modest upregulation following treatment, particularly with vorinostat, though the effect did not reach statistical significance (*Mef2c* RS vorinostat: 37%; RS Rodin‐A 33%; *Pgc1a* RS vorinostat 21%, Figure 8A). GLUT4, a protein whose expression is governed by the same axis [36], followed a comparable pattern (RS vorinostat: +29%, Figure 8B). Since metabolic efficiency and protein turnover are strictly interdependent in dystrophic muscle, we sought to determine whether the restoration of the LKB1 axis also impacted the expression of key autophagy‐related genes. We then evaluated the expression of *Atg12*, a critical mediator of the ubiquitin‐like conjugation system required for autophagosome elongation and maturation (Figure 8A). In D2 *mdx* mice, we found that *Atg12* levels were significantly reduced compared to WT controls (Student's *t*‐test, *p* = 0.023), indicating a compromised basal autophagic capacity, also described in literature [37]. Interestingly, treatment with vorinostat induced a trend toward upregulation of *Atg12* (RS 43%), mirroring the nonsignificant patterns observed for *Mef2c* and *Pgc1a*, whereas this effect was not observed in the Rodin‐A group. To disambiguate whether these effects were attributable to direct HDAC inhibition or to the restoration of LKB1 kinase activity per se, we next evaluated the phosphorylation of SIK1—rather than AMPK, which is constitutively hyperphosphorylated in *mdx* mice [6, 10, 11]—a direct and specific substrate of LKB1 (Figure 8C). SIK1 phosphorylation appeared to be reduced, although not significantly, in D2 *mdx* animals versus D2 WT and remained unaffected by either treatment, suggesting that the restored LKB1 protein did not acquire full signaling competence under the experimental conditions tested.

### 3.6 Limited Benefit of HDACi‐Induced LKB1 Restoration on In Vivo Muscle Performance and CK Levels in D2 *Mdx* Mice

**FIGURE 9:**
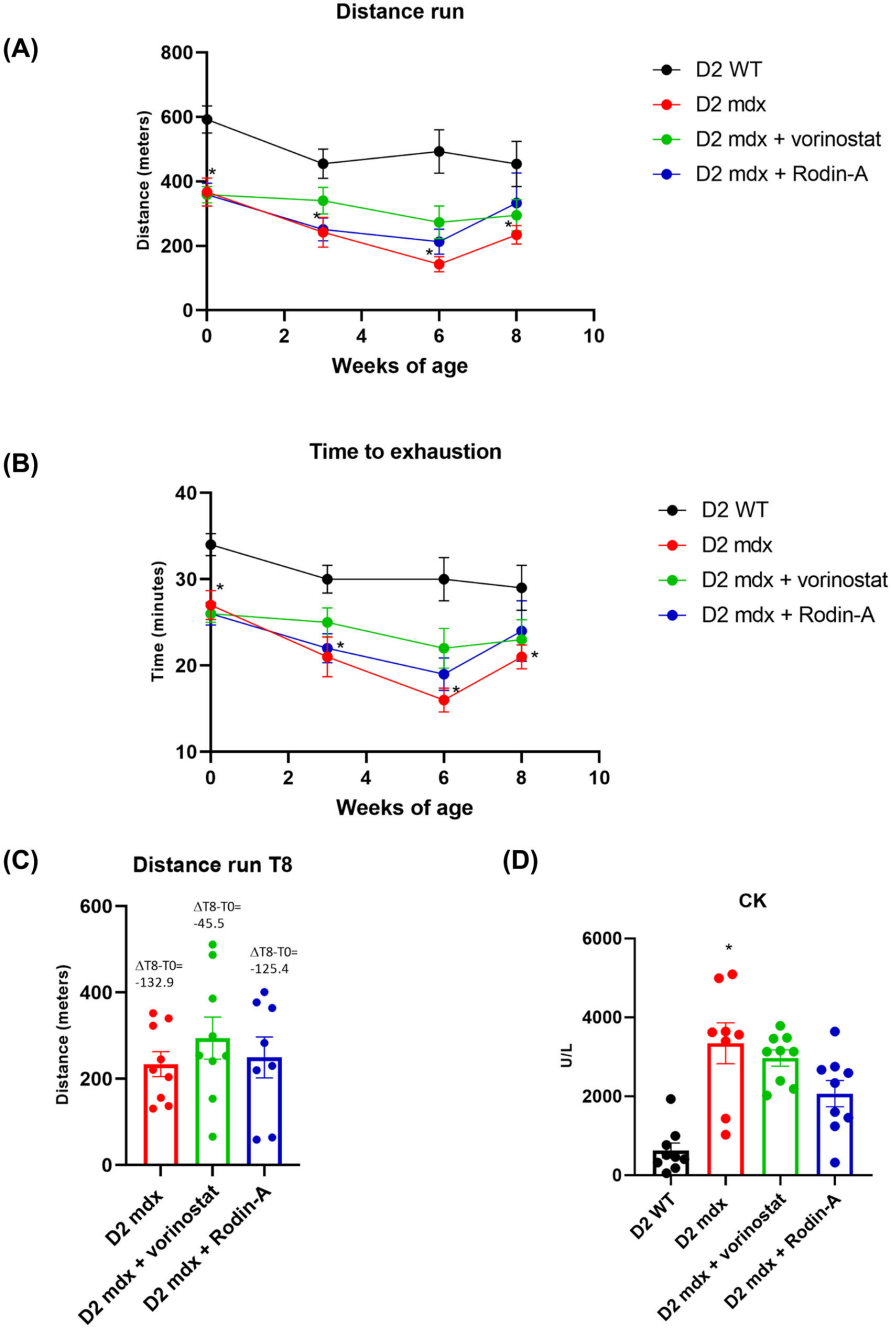
In vivo muscle performance and CK levels in D2 *mdx* mice. All values are expressed as mean ± SEM. A statistically significant difference was found via an unpaired Student's *t*‐test for D2 *mdx* versus D2 WT mice (A: 0.0006 < *p* < 0.01; B: 0.0002 < *p* < 0.05; C: no significant differences among groups were observed; D: *p* = 0.0001). CK, creatine kinase.

To explore the potential relationship between HDACi‐induced LKB1 restoration and in vivo muscle functional performance, we performed an exhaustion test during treatment with HDACi to assess fatigue resistance (Figure 9A–C). Both distance run and time to exhaustion were significantly reduced in D2 *mdx* mice compared to WT controls Student's *t*‐test, A: 0.0006 < *p* < 0.01; B: 0.0002 < *p* < 0.05), consistent with the established exercise intolerance characteristic of the dystrophic phenotype [6, 7, 19]. Vorinostat‐treated mice displayed a nonsignificant trend toward improved performance, which was most clearly revealed when examining the delta in distance run between T8 and T0. In particular, a progressive decline in running distance over time was observed in both untreated and Rodin‐A‐treated dystrophic mice, while vorinostat‐treated animals remained substantially stable, suggesting a preservation of functional capacity rather than a recovery per se.

We also measured plasma CK levels at T8 as a circulating marker of muscle damage (Figure 9D). As expected, CK levels were significantly elevated in D2 *mdx* mice relative to controls (Student's *t*‐test *p* = 0.0001), in line with the sarcolemmal fragility and myofiber necrosis that characterize dystrophin deficiency and that is similarly observed in DMD patients [2]. Notably, CK levels were reduced in both treatment groups, with the effect being more pronounced in Rodin‐A‐treated animals (RS 53%),

### 3.7 LKB1 Expression Decreased During Myogenesis in Patient‐Derived Satellite Cells

**FIGURE 10:**
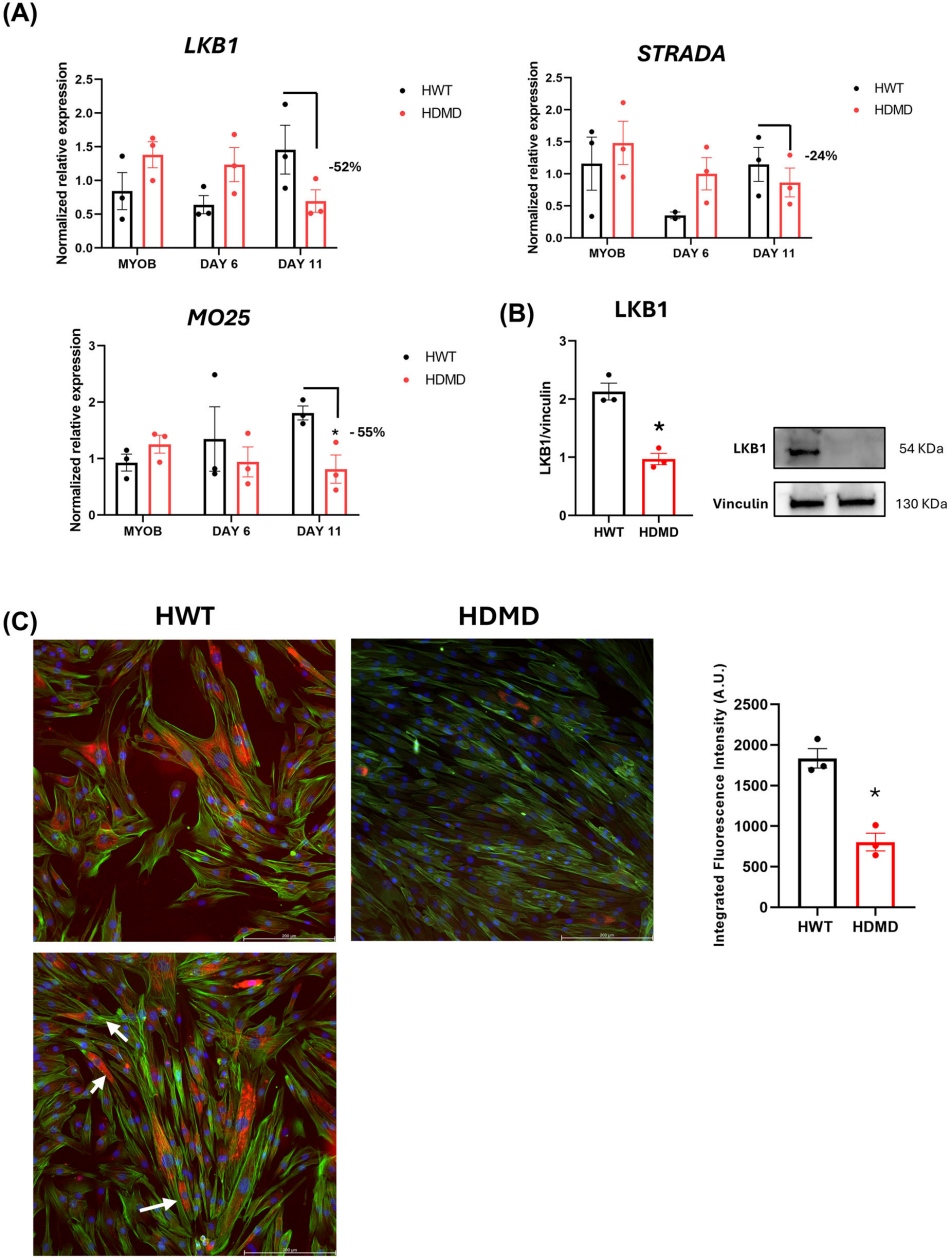
LKB1–STRADα–MO25 expression in patient‐derived dystrophic cells. (A) Gene expression analysis of *LKB1–STRADA–MO25* in myoblasts (D0), and myocytes (D6–D11). Significant differences were assessed by unpaired Student's *t*‐test (MO25, Day 11, vs. D2 WT *p < 0.03). On Day 11 of differentiation, the percent decrease in the expression of *LKB1*, *STRADA*, and *MO25* in HDMD mice compared to HWT (−52%, −24%, and −55%, respectively) is shown in the graphs. (B) LKB1 protein expression quantified by western blot analysis in patient‐derived myotubes (HWT/HDMD, D11). Significant differences were assessed by unpaired Student's *t*‐test (*p < 0.003). (C) Immunofluorescence imaging for LKB1 (20× magnification). D11 HWT/HDMD cells were stained with Hoechst for nuclei staining (blue), LKB1 (red), and phalloidin for the cytoskeleton (green). A significant difference in immunofluorescence intensity signal was found by unpaired Student's *t*‐test (*p = 0.0032). The bottom left panel displays white arrows highlighting the asymmetrical fluorescence of LKB1 signal in HWT myotubes.

To further understand the role of the LKB1 complex in the pathology of DMD, we assessed for the first time its expression in DMD patient‐derived muscle precursors and during the differentiation process. The results of the gene expression analysis of the *LKB1–STRADA–MO25* complex in human myoblasts and myotubes are shown in Figure 10A. In HWT cells, a progressive increase in *LKB1* expression was observed during myogenesis, reaching a peak at Day 11, corresponding to the late stage of differentiation. In contrast, HDMD cells exhibited an opposite trend, with a progressive decrease in *LKB1* expression. Specifically, on day 11, *LKB1* expression was reduced by approximately 50% compared to the myoblast stage and was half of that compared to the level observed in HWT cells at the same time point. Western blot analysis confirmed the significant downregulation of LKB1 at D11 of differentiation in HDMD myotubes compared to HWT (Figure 10B), supported also by the IF conducted at the same time point (Figure 10C). In fact, quantitative IF analysis showed a significant reduction in LKB1 integrated fluorescence intensity in HDMD versus HWT cells. Interestingly, qualitative analysis of LKB1 IF in a subset of HWT cells revealed an asymmetric distribution of the protein, with the fluorescent signal appearing predominantly localized to one side of the myotube (Figure 10C). This asymmetric pattern might be consistent with the well‐established role of LKB1 in cell polarity [38, 39]. Gene expression analysis of *MO25* showed a similar pattern to that of *LKB1* (Figure 10A); dystrophic cells also exhibited a progressive decrease in *MO25* expression over time, and on day 11, it was significantly downregulated if compared to HWT cells (−55%).

The results for *STRADA* showed a less linear trend in HWT cells, with a decrease in gene expression at day 6 followed by an increase at day 11. In HDMD cells, a progressive decrease over time was detected, resulting in a final expression level that was 24% lower compared to that observed in HWT cells.

**FIGURE 11:**
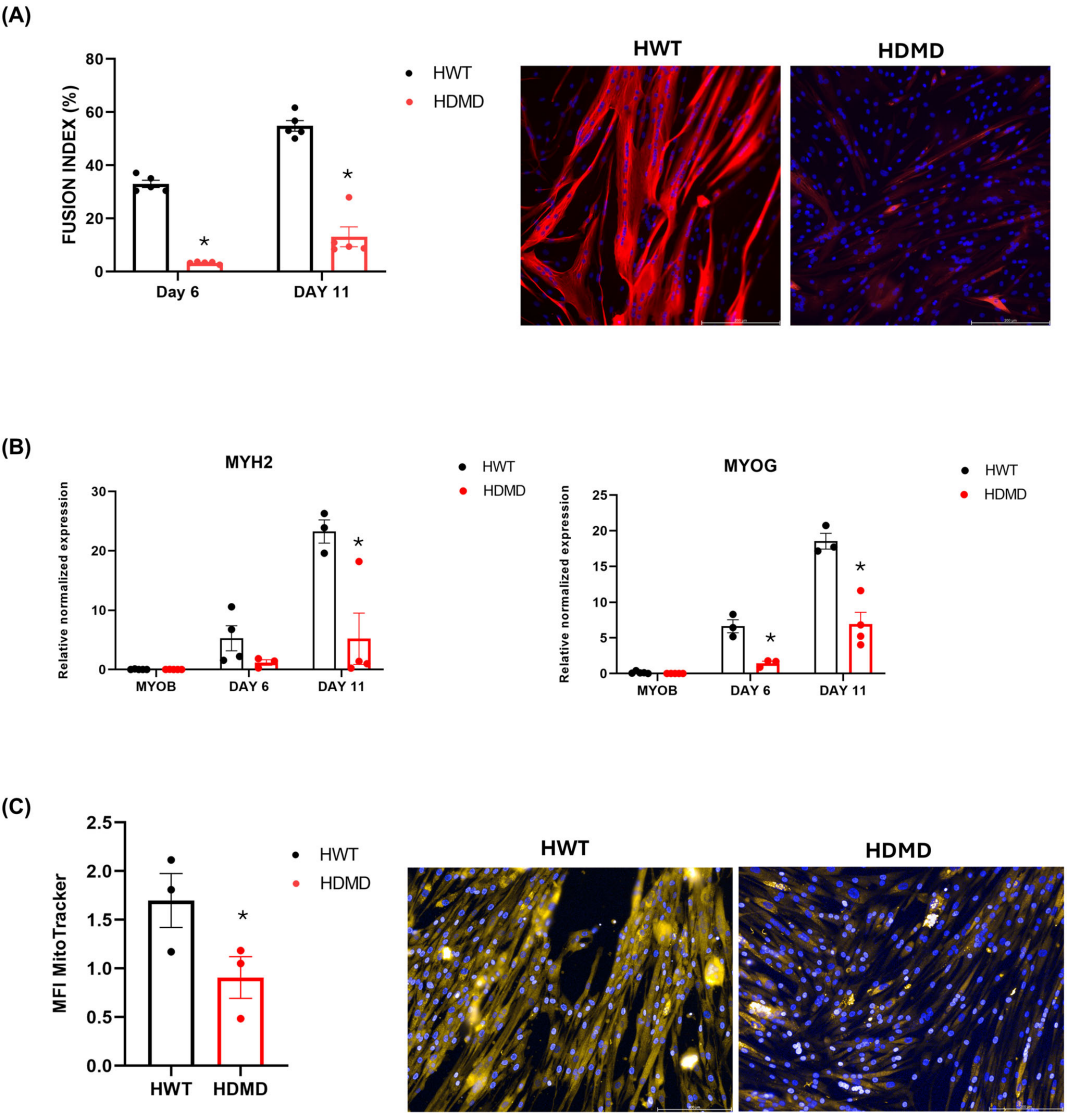
(A) Fusion index (%) for each region of interest on Days 6 and 11 of differentiation. Representative images of MF20 stainin*g* (in red) are shown in A (20× magnification). All data are presented as mean ± SEM. A significant difference was assessed by an unpaired Student's *t*‐test (*p < 0.0001). (B) Gene expression of myogenic differentiation markers (MYOG and MYH2) in HWT and HDMD cell lines at different time points (0, 6, and 11 days). All conditions are from *n* = 3–5 batches. Data are expressed as mean ± SEM. A significant difference was assessed through an unpaired Student's *t*‐test (*p < 0.0001). (C) Quantification of MitoTracker fluorescence intensity normalized to the total number of nuclei for each well in HWT and HDMD myotubes at Day 11 of differentiation. Representative images of MitoTracker staining (in orange) are shown in C (20× magnification). All data are expressed as mean ± SEM. A significant difference was assessed by an unpaired Student's *t*‐test (*p < 0.01).

HDMD cells showed reduced myogenic efficiency compared to HWT at 6 and 10 days of differentiation, as assessed by fusion index analysis (Figure 11A) and qRT‐PCR (Figure 11B). Specifically, the gene expression of late‐stage differentiation markers *MYH2* and *MYOG* was significantly reduced in HDMDs compared to HWT cells. These alterations were confirmed with high‐content imaging for mitochondria using MitoTracker Orange dye (Figure 11C) to evaluate changes in mitochondrial network morphology and fragmentation. Our results showed a significantly lower content in active mitochondria in 11‐day differentiated HDMD compared to HWT.

## 4 Discussion

The findings of this paper support and extend previous work by Boccanegra et al., providing novel and clear insight into the role of LKB1 in dystrophic progression, also by demonstrating a novel potential mechanism of action of HDACi. First, we investigated the regulation of the *Lkb1–Strada–Mo25* complex in skeletal and cardiac muscles across different mouse models of neuromuscular diseases, with a particular focus on ALS and DMD. Furthermore, we evaluated their potential regulation by HDAC inhibition in dystrophic conditions and explored the involvement of regulatory miRNAs in this process.

Our data demonstrate that the expression of the *Lkb1–Strada–Mo25* complex is not altered in the skeletal muscle of SOD1G93A mice, a well‐established model of ALS, suggesting that the dysregulation of this signaling axis is not involved into the pathophysiology of this particular neurodegenerative condition. In contrast, in confirmation of disease‐specificity, a marked downregulation of *Lkb1*, *Strada*, and *Mo25* has been observed in two different murine models of DMD, the BL10 *mdx* and the D2 *mdx* mouse. This was evident in the GC muscle at all ages analyzed, supporting the hypothesis that the LKB1 pathway is broadly and persistently compromised in dystrophic muscle tissue throughout lifespan. The DIA muscle displayed a more selective reduction, with *Lkb1* being the only downregulated component, although not in a statistically significant manner. This may reflect muscle‐specific regulatory mechanisms or a differential sensitivity to pathological stimuli, due to the different workload and type of stimulation to which the DIA muscle is subjected compared to hindlimb muscles, which may result in a lesser involvement of the LKB1–AMPK axis.

Cardiac impairment is a major cause of morbidity and mortality in DMD, and our findings extended the relevance of LKB1 dysregulation to this muscle. *Mdx* mice exhibited substantial reductions in *Lkb1* expression in cardiac tissue with a progressive decline throughout lifespan compared to WT counterpart. Interestingly, the D2 genotype displayed lower expression levels than the BL10 genotype: even D2 WT mice showed reduced *Lkb1* levels, consistent with prior reports that this strain exhibits mild cardiac abnormalities [12]. These findings suggested that LKB1 downregulation may not only be a consequence of dystrophic pathology but could also reflect a predisposition toward general cardiac dysfunction. Accordingly, studies have demonstrated that LKB1 is essential for proper cardiac metabolic function and that cardiac‐specific deletion of LKB1 was associated with the development of hypertrophy and ischemic events [40, 41].

**FIGURE 12:**
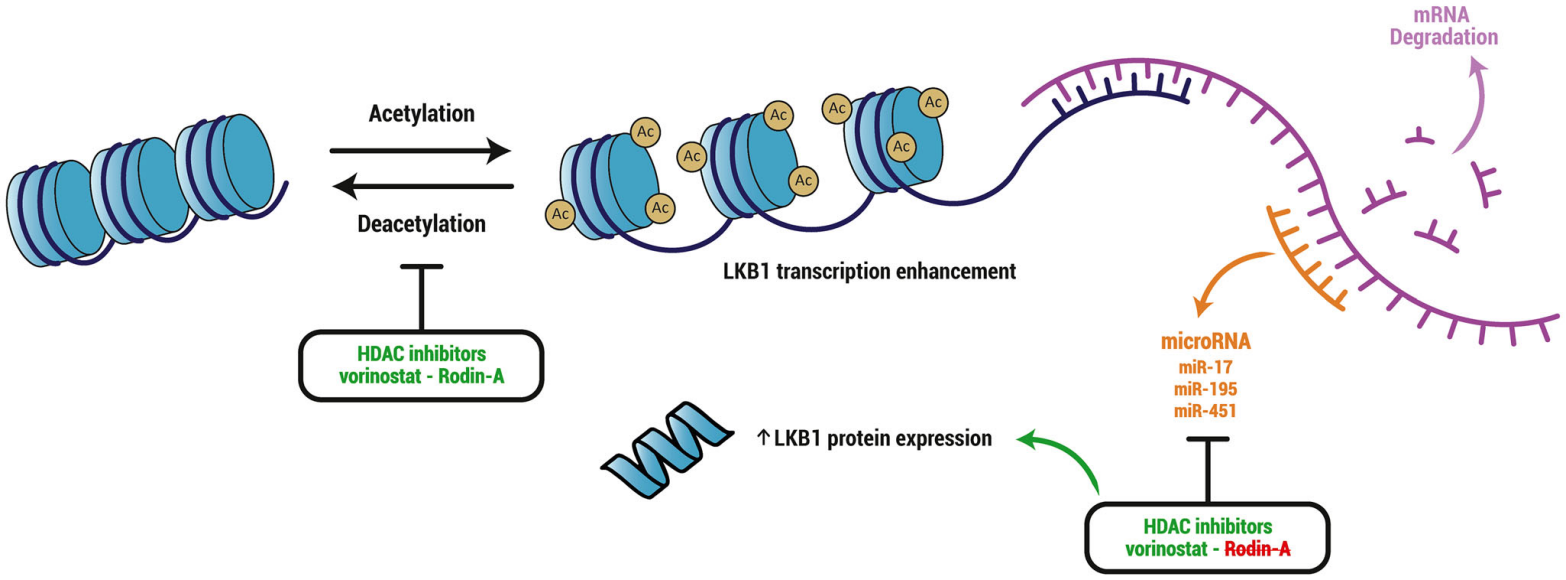
Schematic summarizing the effects of HDAC inhibitors vorinostat and Rodin‐A on LKB1 transcriptional and translational regulation. In dystrophic muscle, vorinostat and Rodin‐A act as HDAC inhibitors and promote histone acetylation to facilitate the transcription of *Lkb1* into mRNA. Vorinostat, but not Rodin‐A, inhibits the expression of microRNAs involved in the degradation of *Lkb1* mRNA (miR‐17, miR‐195, miR‐451), thereby promoting protein translation and increasing its expression.

In addition, we presented a novel finding that expands the current knowledge of the mechanism of action of HDACi (Figure 12). The administration of HDACi significantly restored *Lkb1* expression, particularly in D2 *mdx* mice treated with the HDACi vorinostat. Although Rodin‐A also increased *Lkb1* and *Strada* mRNA expression, its effect did not translate into a robust protein‐level response. Rodin‐A is described as a COREST‐complex selective inhibitor [42], a transcriptional regulator known to recruit HDACs 1 and 2, but not HDAC3 [43]. Rodin‐A also inhibits HDAC activity associated with other HDAC1 and 2 recruiting complexes, but not of HDAC3, as described in the literature [44, 45], suggesting that this compound should be considered as HDAC1 and 2 selective, rather than COREST‐selective. This discrepancy observed between the two HDAC inhibitors is likely related to their different HDAC subtype selectivity. In fact, vorinostat inhibits all class I HDACs in addition to HDAC6, pointing to a possible involvement of HDACs other than HDACs 1 and 2 in this process.

Moreover, our data offered insights into the complex post‐transcriptional regulation of LKB1 expression, focusing on a panel of microRNAs—miR‐451, miR‐195, and miR‐17‐ involved in the post‐transcriptional repression of the kinase. In agreement with our hypothesis, miR‐451 and miR‐195 were indeed upregulated in GC muscles of D2 *mdx* mice versus their D2 WT counterpart, thus emerging as potential contributors to the repression of LKB1 expression. It is known that miRNAs are involved in the modulation of multiple target genes, which makes the assessment of their specific mechanism of action somewhat complex. miR‐451 is upregulated in several inflammatory disorders, as well as in cardiovascular diseases such as heart failure and left ventricular hypertrophy [46]. Given that inflammation is one of the major pathological pathways involved in dystrophic muscle degeneration [4], its upregulation in the D2 *mdx* mouse model appears consistent. Additionally, miR‐195 is involved in the modulation of cellular proliferation and apoptosis; moreover, its upregulation appears to be associated with the inhibition of telomere elongation in satellite cells, thereby promoting their senescence [47]. Its elevated levels in dystrophic muscles may therefore be linked to the reduced replicative capacity of satellite cells, as telomere shortening has been observed in both patients and murine models of dystrophy [48]. In contrast, miR‐17 is only slightly increased in our dystrophic samples (+16%). The miR‐17/92 cluster is among the most extensively studied noncoding RNAs due to its broad implications for human health. Indeed, miR‐17 is overexpressed in a variety of human pathologies, including chronic myeloid leukemia, several types of cancer, cardiovascular disorders, and neurodegenerative diseases [49]. The slight increase in dystrophic muscles observed in our work may be related to the disease stage being analyzed (12 weeks, an acute phase), and further experiments are needed to confirm whether its expression is altered in later stages of the pathology.

Notably, vorinostat treatment was associated with a statistically significant downregulation of all these miRNAs in D2 *mdx* mice muscles, further supporting its role in reactivating LKB1 expression via dual transcriptional and post‐transcriptional regulation. The exact mechanism by which HDACi can modulate miRNA expression remains to be fully elucidated. It is well established that miRNA levels can rapidly fluctuate in response to HDACi treatment, showing both upregulation and downregulation [50]. Some studies have shown that the use of HDACi induces the downregulation of the miR‐17/92 cluster in an in vitro model of colorectal cancer [51], as well as of miR‐451 in patients with renal cell carcinoma when administered in combination with bevacizumab [52]. In contrast, Rodin‐A administration did not show any effect on the expression of the miRNAs of interest. This is likely due to the already described difference in subtype selectivity and suggests the fact that the HDACs specifically inhibited by Rodin‐A are not directly involved in the regulation of the energy stress response in muscle fibers (as is the case for class II HDAC enzymes), but rather in the modulation of the myogenic program and autophagy [53]. These findings may also account for the observation that Rodin‐A restored *Lkb1* expression but not LKB1 protein levels, suggesting that its effects were confined to the transcriptional regulation without influencing post‐transcriptional or translational mechanisms.

The results obtained for MO25 further support this hypothesis. The direct silencing of MO25 by miR‐17, miR‐451, and miR‐195 has indeed been demonstrated and described in the literature [54, 55]. Also in this case, a partial restoration of protein expression is observed only in dystrophic muscles treated with vorinostat, further confirming that Rodin‐A does not affect the post‐transcriptional regulation of the targets under investigation. Future studies employing antagomir‐mediated inhibition of miR‐451, miR‐195, and miR‐17 are needed to establish a causal relationship between these miRNAs and LKB1 regulation in dystrophic muscle and to evaluate their potential as independent therapeutic targets.

Meanwhile, it was pivotal to investigate whether HDACi‐induced LKB1 restoration translates into activation of its downstream signaling cascade. Interestingly, we found that key effectors of the LKB1–Class IIa HDAC axis, such as *Mef2c*, *Pgc1a*, and GLUT4, were only modestly and nonsignificantly upregulated following HDACi treatment, paralleled by a trend in amelioration of *Atg12* expression, as a marker of autophagy‐lysosomal pathway. These results, combined with the absence of any effect on SIK1 phosphorylation and the comparatively limited impact on the accessory proteins MO25 and STRADα, suggest that the heterotrimeric complex required for cytoplasmic retention and full activation of LKB1 may not have been efficiently reconstituted, potentially accounting for the incomplete downstream signaling response. In line with this hypothesis, these molecular trends translated into minor improvements in CK levels and in functional outcomes. Specifically, exhaustion tests on the treadmill revealed only slight stabilization of performance in vorinostat‐treated vs untreated D2 *mdx* mice.

To further strengthen the translational relevance of our work, we analyzed the gene expression of the *LKB1–STRADA* *–MO25* complex in DMD patient‐derived cells during myogenesis, comparing it to that observed in cells derived from a healthy donor. In the latter, *LKB1* expression increases throughout the course of myogenesis, reaching its peak on Day 11. This finding, previously observed also in murine muscle cells [11], was consistent with the role proposed for LKB1 in myotube differentiation [56].

In fact, it has been shown that during myogenesis, microtubules undergo a phase of destabilization that is essential for their subsequent reorganization into the linear arrangement observed in mature myotubes. A 2012 study by Mian et al. demonstrated that LKB1 plays a role in this microtubule destabilization/reorganization, supporting its contribution to the muscle cell differentiation process [56]. Moreover, LKB1 is known to play a key role in the process of cell polarization, which is crucial for cytoskeletal remodeling and, in turn, for proper cellular function [57]. Correct polarization is also essential for the asymmetric division of satellite cells in muscle tissue, a process known to be impaired in dystrophic muscles [4, 58]. Notably, in DMD cells, we observed a reduction in the expression of LKB1, both at the gene and protein level, as well as *Strada* and *Mo25*, during myogenesis, indicating a dysregulation of the complex during the differentiation process. IF analysis further supported this evidence, also showing nuclear confinement of the kinase, as previously observed [11], supporting the hypothesis of LKB1 mislocalization in dystrophic conditions.

## 5 Conclusion

Taken together, our findings highlighted for the first time, that the LKB1–STRADα–MO25 complex is a regulatory node in the pathophysiology of DMD, with consistent dysregulation observed across multiple dystrophic muscle types and disease models. Interestingly, vorinostat treatment clearly restores LKB1 expression in dystrophic muscle, an effect supported both at the protein level and upstream, through the modulation of the miRNAs network that governs *Lkb1* expression. Vorinostat also appeared to be the more effective HDACi in partially restoring functional and metabolic parameters. However, it remains to be elucidated whether these effects are directly driven by the restoration of LKB1 signaling or represent a primary consequence of HDAC inhibition, as both mechanisms converge on the same transcriptional targets. Comprehensive physiological studies are warranted to establish a definitive causal relationship between LKB1 pathway restoration and its long‐term impact on disease progression.

## Author Contributions

**Brigida Boccanegra**: conceptualization, methodology, validation, formal analysis, investigation, data curation, Writing – original draft, Writing – review and editing, visualization, project administration, funding acquisition. **Lisamaura Tulimiero**: methodology, validation, formal analysis, investigation, data curation, writing – original draft, writing – review and editing, visualization. **Raffaella Quarta**: investigation. **Elena Conte**: formal analysis, investigation, data curation, visualization. **Simonetta Andrea Licandro**: investigation, writing – review and editing, supervision. **Alessandra Decio**: investigation, writing – review and editing. **Roberta Lenti**: investigation. **Alberto Ladisa**: investigation. **Giorgia Dinoi**: investigation. **Gaia Carbone**: investigation. **Letizia Claudione**: investigation. **Giulia Maria Camerino**: investigation. **Sabata Pierno**: resources. **Paola Mantuano**: visualization. **Ornella Cappellari**: supervision. **Gianluca Fossati**: resources, writing – review and editing. **Christian Steinkühler**: resources, writing – review and editing, supervision. **Annamaria De Luca**: conceptualization, methodology, validation, resources, writing – original draft, writing – review and editing, supervision, project administration, funding acquisition.

## Conflicts of Interest

The authors declare no conflicts of interest.
